# ARV-based HIV prevention for women – where we are in 2014

**DOI:** 10.7448/IAS.17.3.19154

**Published:** 2014-09-08

**Authors:** Timothy D Mastro, Nirupama Sista, Quarraisha Abdool-Karim

**Affiliations:** 1 FHI 360, Durham, North Carolina, USA; 2 CAPRISA, Durban, South Africa

**Keywords:** HIV, AIDS, antiretroviral agents, women, HIV prevention, HIV treatment, PrEP

## Abstract

Women continue to be at special risk for HIV acquisition due to a complex mix of biological, behavioural, structural, cultural and social factors, with unacceptable rates of new infection. Scientific advances over the past decade have highlighted the use of antiretroviral (ARV) drugs as pre-exposure prophylaxis (PrEP) to prevent HIV acquisition (sexually, parenterally and vertically) and ARV treatment (ART) for HIV-positive patients to prevent onward transmission (treatment as prevention – TasP). This paper reviews the evidence base for PrEP and TasP, describes new products in development and the need to translate research findings into programmes with impact at the population level.

## Introduction

In the fourth decade of the HIV epidemic, the control of HIV transmission remains a global challenge – in 2012, there were an estimated 2.3 million new HIV infections globally [1]. Women continue to be at special risk for HIV acquisition due to a complex mix of biological, behavioural, structural, cultural and social factors [1]. Young women bear a disproportionate burden of HIV infection, notably in sub-Saharan Africa, home to about 70% of new infections [1]. Over the past decade, numerous prospective studies of African women in communities with high HIV infection risk have documented annual HIV incidence rates of 4 to 9%, despite the use of the best available HIV prevention methods [2–4]. A better understanding of the factors contributing to this high HIV risk is needed to guide the use of available HIV prevention tools and to design improved modalities.

Scientific advances in recent years have highlighted the use of antiretroviral (ARV) drugs as pre-exposure prophylaxis (PrEP) to prevent HIV acquisition (sexually, parenterally and vertically) and ARV treatment (ART) for HIV-positive patients to prevent onward transmission (treatment as prevention – TasP). Clinical trials have added greatly to our body of knowledge and several positive findings have raised hopes that effective use of ARVs for prevention, along with other prevention tools, can lead to control of the epidemic. However, programmatic implementation and scale-up are needed to translate these encouraging findings into population level impact. This review provides a synopsis of the scientific evidence for the use of ARVs for prevention, lessons learned from the conduct of these trials, the current state of implementation of ARV-based prevention interventions and an overview of the next generation of ARV-based products for HIV prevention.

## The evidence

### Pre-exposure prophylaxis

Research efforts over the past decade have yielded extensive evidence on ARVs for HIV prevention in women (Table 1). In 2010, the CAPRISA 004 clinical trial of 1% tenofovir vaginal gel demonstrated a 39% level of protection for women in KwaZulu-Natal, South Africa, the first study to show that a vaginal microbicide and an ARV-based product could prevent HIV infection [2]. Since then, we have seen a growing body of evidence of the prevention success of oral tenofovir-based PrEP, either tenofovir disoproxil fumarate (TDF) alone or in combination with emtricitabine (FTC) [5–7]. Daily oral PrEP provided protection for women in serodiscordant partnerships in Kenya and Uganda in the Partners PrEP study (efficacy of 75% for FTC/TDF and 67% for TDF; similar for women and men) [6] and for women in Botswana in the Centers for Disease Control and Prevention (CDC) TDF-2 study (62% efficacy for FTC/TDF among women and men) [7]. Clinical trials also showed that tenofovir-based oral PrEP can be effective in decreasing HIV incidence among men who have sex with men (MSM) [5], and in people (80% men) who inject drugs in Bangkok, Thailand [8].

**Table 1:** Effectiveness studies for ARV-based HIV prevention^a^

Study name	Location	Population	Product	Efficacy (CI)	Adherence estimates^c^	Notes	Adherence intervention
CAPRISA 004 [2]	S. Africa	889 women	Peri-coital vaginal TFV gel^b^	39% (6–60%)	51%	Efficacy was 54% among highly adherent (used gel in >80% of sex acts)	Monthly adherence counselling, applicator count and self-reported coital frequency
iPrEx [5]	North and South America, South Africa, Thailand	2499 men or transgender women who have sex with men	Once-daily oral FTC/TDF b	44% (15–63%)	51%	For first 8 weeks, self-reported pill use was slightly lower in the FTC/TDF arm than in placebo. Afterwards, they were similar, each with a mean of 95%. Rate of pill use, according to pill count, was also similar after week 8; the median ranged from 89–95%. Dispensation dates and quantities of pills suggest pill use decreased from a rate of 99% in the first year to 91% at study conclusion	Monthly adherence counselling, medical history, pill counts
Partners PrEP Study [6]	Kenya, Uganda	4747 heterosexual serodiscordant couples	Once-daily oral FTC/TDF Once-daily oral TDF b	75% (55–87%) 67% (44–81%)	81% 83%	97% of dispensed pills were taken. Study drugs were in use in an estimated 92.1% of total follow-up time	Monthly adherence counselling, sexual behaviour assessment and pill counts
CDC 4940 (TDF2) [7]	Botswana	1200 young men and women	Once-daily oral FTC/TDF	62% (22–84%)	81%	Drug efficacy was highest among highly adherent (those who had taken medication in the past 30 days). Similar rates were found in the intervention and control groups. (Pill counts were 84.1% in FTC/TDF and 83.7% in placebo. Self-reported use was 94.4% and 94.1%, respectively.)	Monthly adherence counselling, self-reported coital frequency and condom use
FEM-PrEP [3]	Kenya, S. Africa, Tanzania	1950 women	Once-daily oral FTC/TDF	6% (−52–41%)	24%	Self-reported data, at discontinuation, indicated 95% of participants had usually or always taken assigned drug. Pill count suggested 88% adherence, yet blood drug level was much lower	Monthly, adherence counselling, self-report, blood samples and pill counts
Bangkok Tenofovir Study [8]	Thailand	2413 men and women who inject drugs	Once-daily oral TDF	49% (10–72%)	66%	Participants chose either 1) daily DOT or 2) monthly visits with study drug diary accounts. They could also switch between these regimens at monthly visits	Monthly adherence counselling, DOT option
VOICE (MTN 003) [4]	S. Africa, Uganda, Zimbabwe	5029 women	Once-daily oral FTC/TDF Once-daily oral TDF Daily vaginal TFV gel	−4% (−49–27%) −49% (−129–3%) 15% (−21–40%)	29% 28% 22%	Adherence estimates are based on a subset of 773 participants. Despite an estimate of 90% adherence (based on self-reported data and pill counts), approximately 30% of participants never had the drug detected	Monthly adherence counselling, self-report and pill counts
HPTN 052 [9]	Malawi, Zimbabwe, Botswana, Kenya, S. Africa, Brazil, Thailand, US, India	1763 heterosexual serodiscordant couples	Early ART (CD4 count of 350–550 cells/mm) vs. delayed ART (CD4 count below 250 cells/mm or development of an AIDS-defining illness)	96% (73–99%)	79% of participants who were in early ART and 74% who were in delayed ART	Adherence to at least 95% study regimen was measured by pill counts	Monthly adherence counselling, self-report and pill counts

a For prevention of sexually transmitted infection

b 1% vaginal tenofovir gel (TFV), oral tenofovir (TDF), oral emtricitabine/tenofovir (FTC/TDF)

c adherence estimates for all but HPTN 052 are based on measuring drug levels from participant samples; estimates for all but the Bangkok Tenofovir Study are from reference [10].

With the exception of the CAPRISA 004 trial that used a coitally linked dosing strategy of tenofovir gel, trials have used a daily dosing strategy. Data from completed PrEP (topical and systemic) trials demonstrate a protective effect of PrEP that ranges from 39 to 75% when adherence is sufficient. However, while ARVs prevent HIV transmission, they have to be used to be effective [10]. In studies that have demonstrated efficacy, there is a clear dose relationship between increased product use and increased levels of protection [11, 12]. The modest effectiveness of 39% in CAPRISA 004 was increased to 54% in those with high levels of adherence [2] and to 74% in women in women with drug levels higher than 1000 ng/ml [13] supporting the importance of both adherence and biological factors impacting efficacy.

In the two trials that did not demonstrate PrEP efficacy, FEM-PrEP and VOICE, while self-reported adherence levels were high, measurement of drug levels indicated low levels of product use [3, 4, 14]. There are likely many factors that impact adherence and these need to be better elucidated to inform future trials and programmatic use. Interviews of women in the FEM-PrEP trial of daily, oral FTC/TDF PrEP indicated that low adherence was linked to perceptions of low risk for HIV acquisition, use of an investigational drug, fear of and/or experience of adverse effects, possible assignment to the placebo, difficulties with and dislike of taking pills and influence of other people [15].

In contrast to treatment trials that have proxy markers of HIV viral levels and CD4+ cell counts for monitoring therapeutic success, HIV prevention trials do not have a correlate of risk or protection other than HIV infection, making monitoring of the use of microbicides or PrEP products challenging. Formulation impacts drug concentrations in the genital tract; topical tenofovir gel used vaginally yields 1000-fold higher concentrations in vaginal fluids compared to oral formulations [13]. While measuring drug levels is an important advance in PrEP studies, there is no benchmark for what levels need to be achieved and sustained to prevent HIV infection. In the ART of HIV-positive persons, maintaining steady states of drug levels is key to success; for uninfected individuals the levels needed are less clearly defined. This is an important opportunity and gap that will need to be addressed as the product pipeline, dosing strategies and formulations for testing expand.

Other concerns raised about the use of PrEP include drug resistance and compromising first-line drug regimens. Thus far, acquired ARV drug resistance has not been a substantial problem in those who have acquired HIV infection while on PrEP. However, inadvertently initiating FTC/TDF PrEP in people who are already HIV positive, especially those with acute infection, has resulted in FTC resistance [5]. On-going follow-up of persons who became HIV positive on PrEP will generate empiric evidence on disease progression and treatment outcomes with TDF/FTC containing first-line drug regimens. To date the safety profile of PrEP regimens has been good, with no major safety concerns identified [3, 5, 16, 17]. PrEP has not interfered with the effectiveness of hormonal contraceptives [3, 18].

In the United States, in 2012, based on available clinical trial data, the Food and Drug Administration (FDA) approved oral FTC/TDF for PrEP for people at high risk of sexual acquisition of HIV. The US CDC subsequently issued PrEP guidance for MSM, heterosexual men and women, and following the trial among people who inject drugs in Thailand [8], added an indication for people who inject drugs. In 2014, WHO issued PrEP guidance as part of consolidated guidelines on HIV prevention, diagnosis, treatment and care for key populations (including female sex workers). Generalized guidelines for women and country-specific regulatory filings are eagerly awaited.

## PrEP offers new hope for women

ARV-based PrEP (systemic and topical) offers a promising female-controlled method of prevention. The outcomes of the FEM-PrEP and VOICE trials highlight the need to know more about individual behaviour, optimal formulations and dosing strategies for acceptability. The central importance of adherence in PrEP success has prompted examination of its determinants [19, 20]. A systematic review of 24 phase II and phase III microbicide trials has highlighted that major reasons for non-adherence include high on-trial pregnancy rates (resulting in withdrawal of study drug), low trial retention rates, low participant perception of risk, migratory partners and trial fatigue [21]. Individual participation characteristics, including young age, may also influence adherence, as well as study product characteristics [21]. Findings from the FEM-PrEP trial suggest that some women enrolled and remained in the study for benefits other than the HIV prevention that might be offered by the study drug, including clinical care; reimbursement for study visits was reported to be a minor contributor [22]. When assessing factors related to adherence in a placebo-controlled clinical trial of a product of unproven efficacy, it is important to remember that different forces will be encountered in the programmatic use of a product proven to be effective. It is critically important to continue to characterize such factors, especially as they pertain to target at-risk populations for PrEP, and to inform the development of new technologies that rely less on adherence.

While PrEP studies to date have not identified serious safety problems, both the FEM-PrEP and VOICE trials reported substantial rates of adverse effects (including nausea and headache) suggesting that these effects may have contributed to women stopping the drugs after initially using them [3, 4]. Very low levels of adverse effects will be important to maximize PrEP uptake and adherence, as users will be otherwise healthy individuals.

Notwithstanding the need to weigh the risks and costs of PrEP and TasP and identify target populations who are likely to benefit most from PrEP, modelling exercises offer compelling estimates on its preventive benefits at a population level. Effective use of PrEP among those at highest behavioural risk could avert 3.2 million new HIV infections in sub-Saharan Africa over the next 10 years [23]; and, in South Africa, broad use of tenofovir gel could avert up to 2 million new infections and 1 million deaths over the next two decades [24]. Oral and topical PrEP can be relatively cost-effective interventions [25, 26]. In light of the continued and urgent need for HIV prevention interventions that work in women and the promise of PrEP for altering epidemic trajectories, a more concerted effort on user and provider needs and preparedness is needed.

In terms of next steps with tenofovir gel, the FACTS 001 trial, designed to confirm the results of the CAPRISA 004 trial, is underway at seven sites in South Africa and is scheduled for completion in 2015. A successful outcome could lead to licensure of tenofovir gel. The CAPRISA 008 follow-on study is simultaneously assessing the feasibility of integrating microbicide provision into family planning services and is currently underway. Draft normative guidance has been prepared by WHO, and the South African government has initiated plans for local product manufacture for public sector distribution. Discussions with the US FDA have provided guidelines of requirements for licensure.

## Next-generation products and trials

We are early in the era of ARVs for HIV prevention and we can expect that additional products and formulations in the research and development pipeline will advance to clinical trials and, hopefully, programmatic use. Alternative new drugs, regimens and formulations for PrEP are needed in order to provide choices to men and women, similar to available choices of contraceptive methods. New drugs will provide an alternative to FTC/TDF, which is efficacious and generally safe but is occasionally associated with increased creatinine levels (indicating renal impairment) and decreased bone density and is associated with side effects (e.g., nausea, headache) that may impact adherence. Furthermore, FTC/TDF is rapidly becoming part of first-line ART for many countries. Orally administered TDF (yielding TFV in plasma) does not concentrate in the cervix or vagina after a single dose and this may reduce “forgiveness” in the protection of women if doses are missed [12, 27], and the emergence of ARV resistance remains a concern. Therefore, PrEP agents that have a higher threshold for resistance and/or in which the emergence of resistance will not impact efficacy of first-line regimens are needed. Moreover, since daily PrEP, in either oral or gel formulation, may be a difficult adherence goal for some healthy individuals, finding alternative dosing regimens is a priority [28]. Therefore, development of long-acting injectable products, vaginal rings and patches is an important undertaking. Such products have the potential to prevent HIV acquisition without relying on adherence to a daily oral or gel insertion regimen.

The next generation of topical microbicides being evaluated includes products that do not require use daily or are linked to sexual events and monthly formulations. Such products could be more convenient to women and could provide long-term protection from HIV infection during unanticipated and anticipated sexual exposures. A vaginal ring that is replaced every four weeks and contains dapivirine (DAP, a non-nucleoside reverse transcriptase inhibitor [NNRTI]) in silicone elastomer is being evaluated in phase III studies (MTN 020 “ASPIRE” and IPM 027 “Ring” trials). The results of these trials are anticipated in 2015. Other rings including those that contain maraviroc (MVA, a CCR5 receptor antagonist), MIV 150 (an NNRTI) and a combination of DAP and MVA are being evaluated in phase I and phase II [29, 30]. Preliminary results indicate that the vaginal rings containing DAP, MVA or a combination of the two drugs are safe and tolerable and DAP, but not MVA, was quantifiable in vaginal tissue [29]. A film formulation of DAP was demonstrated to be safe with measureable levels of drug in plasma and tissue [31]. Other products, including a ring containing a combination of DAP and darunavir and a vaginal tablet containing TFV and FTC, are being evaluated for efficacy in preclinical studies [30].

An advantage of these long-term dosing formulations, in addition to the lack of dependence on daily adherence, is the ability to combine agents for other indications such as contraceptives or drugs for treatment of other sexually transmitted infections. Preclinical studies are underway to evaluate combinations of the contraceptive hormone levonorgestrel with either DAP or TFV [32]. Injectable agents are already being used by women for contraception. In the future, an effective PrEP agent has the potential to be combined with an effective contraceptive for prevention of both HIV infection and pregnancy. Such multi-purpose technologies formulated either as an injectable formulation or in a vaginal ring would be a valuable tool for HIV prevention in women in resource-limited settings, including sub-Saharan Africa where the majority of the world's HIV-positive women reside.

Two injectable ARV agents currently in development are TMC278LA, an injectable formulation of rilpivirine (an NNRTI), and GSK1265744, an integrase inhibitor [33, 34]. TMC278LA is a novel poloxamer 338–containing formulation of TMC278. TMC278LA is long-acting and well-suited for delivery via intramuscular injection and is currently being considered for both ART and PrEP. Studies have demonstrated the safety of oral rilpivirine, which is approved by the US FDA for treatment as well as the safety of single doses of TMC278LA. The long-acting injectable formulation is also being developed for treatment of HIV infection. Initial studies have demonstrated the safety of single doses of TMC278LA in men and women [35]. The safety of long-term dosing of TMC278LA for PrEP will be evaluated in HIV-negative women in the United States, South Africa and Zimbabwe in the HPTN 076 study (www.hptn.org).

Another injectable agent, GSK1265744, is an investigational HIV-1 integrase strand transfer inhibitor that possesses attributes favourable for both HIV treatment and PrEP indications. In macaques, single, monthly injections of GSK744 LA that replicate the human dose were fully protective against repeated vaginal SHIV exposures [36]. GSK744 LA also prevented rectal SHIV transmission in macaques [37]. These data support the advancement of GSK744 LA as a PrEP candidate for women. It is also currently in phase II clinical trials evaluating the efficacy and safety for treatment of HIV infection [33]. The safety of GSK744 LA as a PrEP agent will be evaluated in men and women at sites in the United States, Brazil and sub-Saharan Africa in the HPTN 077 study (www.hptn.org). Both TMC278 LA and GSK744 LA have a pharmacokinetic profile that allows monthly to trimonthly parenteral dosing using a nanosuspension formulation. Another injectable agent, an investigational monoclonal antibody, Ibalizumab binds to the primary receptor for HIV, CD4, and inhibits viral entry [38, 39]. It is being evaluated for the treatment of HIV infection in combination with an optimized background regimen, and it may also show promise in its utility as a PrEP agent [34].

## Treatment as prevention

The HPTN 052 clinical trial of early (CD4 cell counts of 350–500 cells/µL) ART compared to delayed treatment for HIV-positive persons with HIV-negative heterosexual partners demonstrated a 96% reduction in transmission [9]. Protection was similar for men and women; 50% of the HIV-positive partners were women [9]. This is the most compelling evidence for the effectiveness of ARVs for preventing HIV infection [9]. Modelling data demonstrate the enormous prevention benefits from implementing these findings especially in settings with a high HIV disease burden [40–43]. Access to HIV testing services and linkage to care and treatment services will be needed to reduce individual viral loads and onward transmission, but there is still uncertainty about the coverage, uptake and compliance rates that will be needed to achieve an AIDS-free generation alone and in combination with other prevention interventions.

In 2013, WHO issued new guidelines for ART that increased eligibility to HIV-positive persons with CD4 cell counts less than 500 cells/µL, all pregnant and breastfeeding women, persons with HIV-negative partners, persons with tuberculosis and hepatitis B virus infection and children less than five years of age [44]. These changes greatly increase the number of people globally who “need” ART and will strain health systems and resources to achieve full or even near-full coverage. Women should benefit from the hoped-for population-level effect on HIV transmission. However, achieving high levels of HIV testing and identifying persons with acute and early HIV infection remains challenging. On-going studies of combination HIV prevention strategies that include early ART for all HIV-positive persons are intended to measure the effect of early ART on population-level HIV incidence (TasP, POP-ART, Botswana). Even with high levels of ART coverage, it is likely that there will continue to be a pressing need for women-controlled prevention methods.

A number of trials and demonstration projects are currently underway to assess the feasibility of implementation and the impact on HIV transmission of TasP at a population level (e.g., HPTN 065 in Washington DC and the Bronx, New York; ANRS/Africa Centre in KwaZulu-Natal, South Africa; POP-ART/HPTN 071 in Zambia and South Africa; and MSF in Malawi, Swaziland and South Africa) [30]. The translation of these trial findings will be influenced by the ability to deliver TasP in settings where AIDS-related stigma and discrimination are prominent, health care delivery systems are weak and the ability to increase coverage of treatment for those who need treatment for individual benefits are limited. Additional concerns include treatment monitoring costs and the development of drug resistance due to poor adherence. Modelling exercises suggest that high levels of ART coverage (>80%) will be needed to substantially decrease population level HIV incidence [45]. However, ecological, observational level data from Hlabisa, KwaZulu-Natal, South Africa, that indicate that a modest 35% coverage of ART reduced HIV incidence by 34% are encouraging [46].

An additional concern about TasP in settings where young women bear a disproportionate burden of HIV infection as a result of age-disparate relationships with male partners who have been reluctant to practice safer sex is the male partners’ level of willingness to initiate early ART for the prevention of infection to their sexual partner. Some of these concerns were raised in considering access to ART in sub-Saharan Africa in the early 2000s and to date have not materialized. Revised HIV treatment guidelines have increased the number of people eligible for ART. With regard to HIV-TB co-infection, in some parts of Africa, HIV-TB co-infected patients account for about 70% of patients using TB services. With the expansion of ART eligibility to these patients, perhaps the issue of when to start ART will become a less controversial issue. With increased implementation and expanded coverage of HIV testing and linkage to care and treatment, those with acute HIV infection will contribute a larger proportion of all new infections. Strategies including laboratory-based assays for identifying these individuals quickly and intervening early will be central to maintaining reductions in onward transmission of HIV and gains made from TasP [20].

## Conclusions

There remains an urgent need to improve and scale-up HIV prevention efforts for women, especially in southern Africa. We have seen that the most effective HIV prevention efforts have employed a combination approach, customized for the specific settings and populations. Providing women with a variety of prevention options will likely lead to the most effective prevention for the most women in the widest range of settings. Such a strategy has been effective in addressing women's needs for contraception. Over the next decade, following new WHO guidelines, we will see an increase in the number of people starting ART at higher CD4 cell counts. This will lead to decreased HIV transmission; however, there will continue to be a pressing need for women-controlled prevention methods as not all male partners will be virally suppressed. The sobering observations of the challenges in many settings of achieving high levels of coverage in the various steps of the HIV prevention and treatment cascade will require new, more effective methods for HIV-negative women. Moreover, there continues to be a pressing need to increase levels of HIV testing to identify HIV-positive persons, including those with acute infection.

On-going and planned studies of new ARV-based prevention methods hold the promise of offering women highly effective prevention that can substantially limit HIV transmission. Studies to date have not identified sex differences in the levels of HIV protection provided by ARV-based methods; the overriding factor for both women and men is the level of adherence to products. It will be important to enhance our understanding of women's perceptions of risk and the complex behavioural dynamics that influence adherence to complement the development of new biologic products. On-going and planned PrEP demonstration studies will add greatly to our understanding of how ARV-based products will add to our HIV prevention tool kit (avac.org) [47]. The technical advances remain an important first step but are no magic bullets. We need to enhance our understanding of the bio-behavioural nexus, and demonstration projects will help identify appropriate target populations, acceptability of products, demand creation strategies and product use in non-trial settings.
